# Patients with low muscle mass have characteristic microbiome with low potential for amino acid synthesis in chronic liver disease

**DOI:** 10.1038/s41598-022-07810-3

**Published:** 2022-03-07

**Authors:** Kenta Yamamoto, Yoji Ishizu, Takashi Honda, Takanori Ito, Norihiro Imai, Masanao Nakamura, Hiroki Kawashima, Yasuyuki Kitaura, Masatoshi Ishigami, Mitsuhiro Fujishiro

**Affiliations:** 1 Department of Gastroenterology and Hepatology, Nagoya University Graduate School of Medicine, 65 Tsuruma-cho, Showa-ku, Nagoya, 466-8550 Japan; 2 Laboratory of Nutritional Biochemistry, Department of Applied Biosciences, Graduate School of Bioagricultural Sciences, Nagoya University, Nagoya, Aichi 464-8601 Japan

**Keywords:** Liver cirrhosis, Translational research

## Abstract

Sarcopenia is thought to be related to the microbiome, but not enough reports in chronic liver disease (CLD) patients. In addition to the differences in microbiome, the role of the microbiome in the gut is also important to be clarified because it has recently been shown that the microbiome may produce branched-chain amino acids (BCAAs) in the body. In this single-center study, sixty-nine CLD patients were divided by skeletal muscle mass index (SMI) into low (L-SMI: n = 25) and normal (N-SMI: n = 44). Microbiome was analyzed from stool samples based on V3-4 region of bacterial 16S rRNA). L-SMI had a lower Firmicutes/Bacteroidetes ratio than N-SMI. At the genus level, *Coprobacillus*, *Catenibacterium* and *Clostridium* were also lower while the *Bacteroides* was higher. Predictive functional profiling of the L-SMI group showed that genes related to nitrogen metabolism were enriched, but those related to amino acid metabolism, including BCAA biosynthesis, were lower. The genes related to 'LPS biosynthesis' was also higher. The microbiome of CLD patients with low muscle mass is characterized not only by high relative abundance of gram-negative bacteria with LPS, but also by the possibility of low potential for amino acid synthesis including BCAAs.

## Introduction

Hepatocellular carcinoma (HCC) and cirrhosis are important prognostic factors in patients with chronic liver disease. A systematic review of sarcopenia showed sarcopenia is also an independent prognostic factor for a worse prognosis, independent of cirrhosis and HCC^1^. It was also reported that sarcopenia increased the risk of infection and prolonged hospital stay. Various factors such as protein-energy malnutrition, lipopolysaccharide (LPS)-mediated systemic inflammation, elevated myostatin, and anabolic resistance are causes of sarcopenia. However, clinically, the prediction of sarcopenia, risk assessment, and sarcopenia mechanisms remain unclear.

In recent years, muscle-microbiome association is recognized as the gut-muscle axis ^2^. The association are also affected by various factors, including butyrate and LPS, which may be anti-inflammatory and pro-anabolic mediators^2,3^. It has also been suggested that the amino acids synthesized by the microbiome are associated with sarcopenia^4^. The patients with chronic liver disease are at risk of ammoniaemia, and the risk is reduced by antibiotics and laxatives. It also may suspect their microbiome tend to use nitrogen for ammonia synthesis instead of muscles.

Lin Kang et al^5^. showed that alpha diversity and relative abundance of Firmicutes were lower in patients with sarcopenia. They also compared predicted genes of microbiome and showed enrichment of LPS biosynthesis. However, it is not a study of patients with chronic liver disease. Patients with chronic liver disease have a characteristic microbiome even if alanine aminotransferase is normal. Ponziani FR et al^6^. compared the microbiome in cirrhotic patients with or without sarcopenia and found low levels of several categories of genes, including enzymes related to BCAA synthesis. Recently, it has become clear that the microbiome is associated with serum BCAAs^4^, and there is a need for more detailed evaluation in clinical practice. We examine fecal samples to characterize the proportions and roles of microbiome in patients classified into the normal skeletal muscle mass index (N-SMI) group and low SMI (L-SMI) group. We also did metabolic mapping based on the Kyoto Encyclopaediaedia of Genes and Genomes (KEGG)^7^^、^^8^^、^^9^ because analysis based on category alone is not enough to assess the metabolic pathway for BCAAs.

## Results

### Patient background

The patient background is listed in Table 1; no significant differences were found between the N-SMI and L-SMI groups with respect to age, weight, and liver function, such as ALBI score and Fib4-index, or in the use of proton pump inhibitors, diabetes, frequency of bowel movements per week, and stool characteristics, which may affect the microbiome. There was no significant difference between the N-SMI group (20.5%) and the L-SMI group (24.0%) in the percentage of patients taking branched-chain amino acid (BCAA). In contrast, the HCV rate was lower in the L-SMI group (52.0%) compared to the N-SMI group (77.3%). Patients responded to questions regarding their dietary habits in a questionnaire. Most patients mainly follow a typically Japanese lifestyle. The questionnaire’s return rate was 68.1% and showed no significant difference in the main intake of meat, fish, and vegetables.

**Table1:** Patient characteristics.

	n	N-SMI group	n	L-SMI group	*p*-value
Gender (Females/males)	44	30/14	25	14/11	0.312
Age (years old)	44	66 (57.5–71.5)	25	68 (62.0–73.3)	0.291
Body weight (kg)	44	162 (155–167)	25	162 (156–167)	0.132
Height (cm)	44	61.5 (57.6–68.6)	25	60.7 (54.4–64.2)	0.866
Etiology (HBV/HCV)	44	10/34	25	12/13	0.030
Alcohol(0/ < 60 g/ < 60 mg/day)	44	29/9/6	23	18/5/0	0.207
Child–Pugh class (A/B/C)	44	36/7/1	25	21/3/1	0.849
Diabetes mellitus (no/yes)	44	29/15	25	19/6	0.381
HCC (no/yes)	44	14/30	25	13/12	0.098
PPI (no/yes)	44	29/15	25	18/7	0.602
UDCA (no/yes)	44	23/21	25	14/11	0.765
BCAA (no/yes)	44	35/9	25	19/6	0.731
Frequency of bowel movement per week (> 8/≦7)	32	13/19	17	4/13	0.236
Hardness of stool (Hard /normal /soft)	32	8/19/4	16	4/10/2	0.078
Exercise habits once a week (no / yes)	32	14/18	16	6/10	0.763
Varix (no/yes)	27	10/17	17	8/9	0.510
Platelet count (10^3^cells/ul)	44	140 (104–186)	25	122 (82–162)	0.333
AST (IU/l)	44	31 (23.5–60.0)	25	42 (22.3–48.5)	0.540
ALT (IU/l)	44	26 (19–46)	25	29 (18–36)	0.707
Albumin (g/dl)	44	4.0 (3.7–4.8)	25	3.8 (3.3–4.2)	0.276
Total bilirubin (mg/dl)	44	0.9 (0.6–1.3)	25	1.2 (0.7–1.6)	0.295
Prothrombin time (INR)	44	1.02 (0.99–1.10)	25	1.07(0.99–1.10)	0.235
HbA1c (%)	26	5.85(5.3–6.9)	13	5.4 (4.95–6.1)	0.132
Creatinine (mg/dl)	44	0.85(0.64–0.96)	25	0.71(0.63–0.87)	0.464
HCV-RNA, (detected/SVR)	25	12/13	10	2/8	0.132
Fib4-index	44	3.09 (2.16–7.24)	25	4.75 (2.36–6.92)	0.680
ALBI score	44	− 2.6( − 2.8 to 2.2)	25	− 2.4( − 2.8 to 1.9)	0.251
SMI (CT:L3 level)	44	47.1 (43.9–50.7)	25	37.2 (35.6–39.7)	< 0.001
CT value (HU)	44	35.2(31.8–39.1)	25	33.6 (25.5–37.0)	0.024
Questionnaire on dietary lifestyle.*					
Main protein intake (Meat/fish)	30	12/18	16	7/9	0.808
Vegetable (enough/ not enough)	31	24/7	16	12/4	0.854
Style (Japanese / Western)	31	23/8	14	11/3	0.754

HCC; Hepatocellular carcinoma, PPI; Proton Pump Inhibitor, UDCA; ursodeoxycholic acid, BCAA; Branched Chain Amino Acid, AST; aspartate aminotransferase, ALT; alanine aminotransferase, HbA1c; hemoglobin A1c, SVR; sustained virological response, Fib4-index; age (years old) × AST (IU/L)/(platelet count (10^9^/L) × √ALT (IU/L)), ALBI score; Albumin-bilirubin score, SMI; Skeletal muscle mass index. HU; Hounsfield unit. *: Patients were given a self-report questionnaire to choose from two options for a more suitable dietary lifestyle.

### Comparison of alpha diversity and relative bacteria abundance in each group

**Figure 1:**
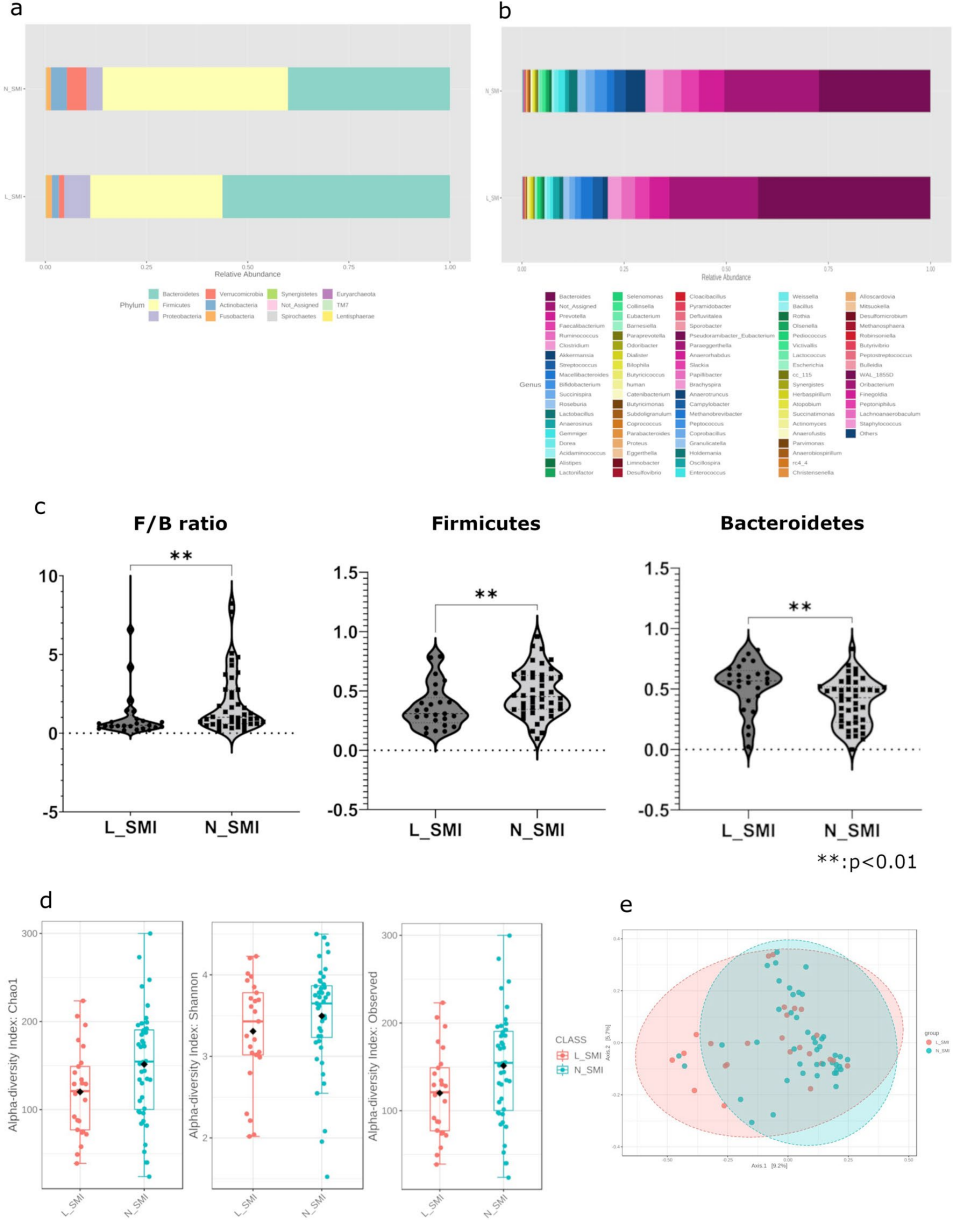
(**a**) Proportions of the microbiome at the phylum level. The N-SMI group had a higher proportion of Firmicutes and a lower proportion of Bacteroidetes compared to the L-SMI group. Firmicutes to Bacteroidetes ratio (F/B ratio) was higher in the N-SMI group compared to the L-SMI group. (**b**) Proportions of the microbiome at the genus level. The phylum Bacteroidetes comprises approximately 90% of the two types of bacteria, *Bacteroides* and *Prevotella*. In contrast, the phylum Firmicutes comprises various types of bacteria such as *Catenibacterium*, *Coprobacillus*, and *Clostridium*. (**c**) F/B ratio and relative abundance of each in the two groups. All two groups were significantly different. F/B ratio (p = 0.0091), Firmicutes (p = 0.0099919, FDR = 0.059951), Bacteroidetes (p = 0.0036818, FDR = 0.044182). One sample in L_SMI contained an outlier of the F/B ratio in the figure. (**d**) Comparison of alpha diversity. Significantly higher values for N_SMI in Observed species (p = 0.029831) and Chao1 (*p* = 0.02936); Shannon index (*p* = 0.2399) was not significantly different. (**e**) Comparison of β-diversity. No significant difference was found between the two groups. (**c.d,e)** : Mann–Whitney U test. < Supplementary information for rare names of genus-level bacteria > human: a member of the Rikenellaceae family of bacteria, which includes the gut metagenome as defined by Greengene 13.8. cc_115: Erysipelotrichaceae family, rc4_4: Peptococcaceae family, WAL_1855D: Tissierellaceae family as defined by Greengene 13.8.

The percentage of bacteria in each group is shown in Fig. 1a at phylum and Fig. 1b at genus level. The microbiome was classified into eleven phylum level bacteria. Bacteroidetes (L-SMI; 56.2%, N-SMI; 40.1%, *p* = 0.004) were significantly higher in the L-SMI group compared to the N-SMI group (Fig. 1c). In contrast, the relative abundance of Firmicutes (L-SMI; 32.7%, N-SMI; 45.8%, *p* = 0.01) was significantly lower in the L-SMI group compared to the N-SMI group. Firmicutes to Bacteroidetes ratio was also significantly lower in the L-SMI group compared to the N-SMI group (*p* = 0.0091).

A total of 176 genus level bacteria were detected. MicrobiomeAnalyst extracted the top 91 genera of bacteria and created a bar graph for each group (Fig. 1b). The bacteria with the highest relative abundance in Bacteroidetes were *Bacteroides* (L-SMI group; 42.3%, N-SMI group; 27.3%). *Prevotella* were found in 4.85% of the L-SMI group, and 6.25% of the N-SMI group. *Bacteroides* were more common in the L-SMI group, and *Prevotella* were more common in the N-SMI group. The relative abundance of the two bacteria in Bacteroidetes was 90.1% in the L-SMI group, and 89.7% in the N-SMI group. In contrast, Firmicutes contained a variety of bacteria.

Alpha diversity (Chao1 and observed species) in the L-SMI group was significantly lower than that in the N-SMI group (Fig. 1d). Shannon index in alpha diversity and beta diversity profiling (Fig. 1e) were not significantly different.

### Comparison of microbiome via linear discriminant analysis effect size (LEfSe; taxonomic comparison)

**Figure 2:**
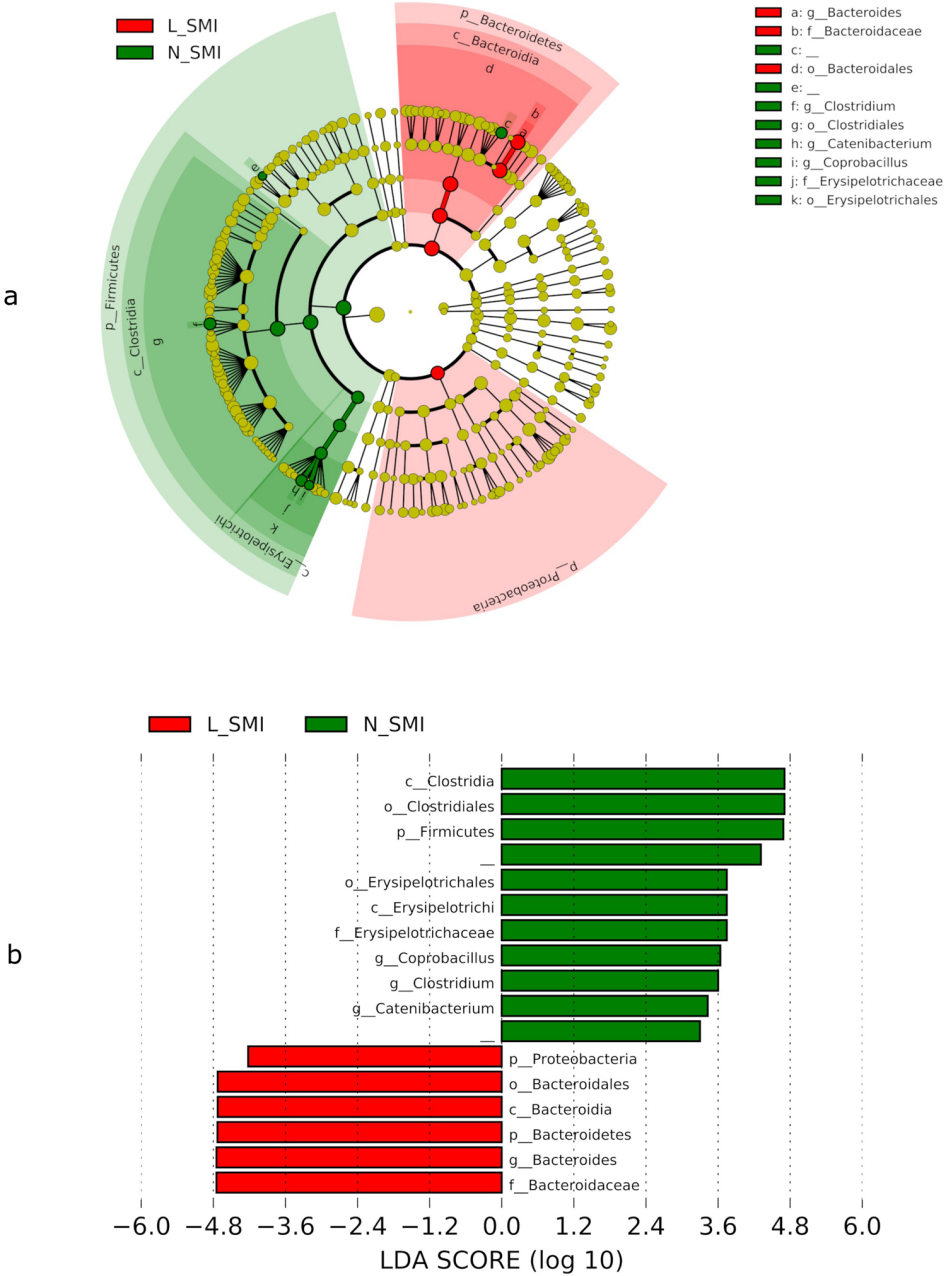
Comparison of the microbiome in the N-SMI or L-SMI group. (**a**). The cladogram of bacteria that showed significant difference between the two groups. The cladogram shows the bacteria marked with green or red bars significantly different between the two groups (*p* < 0.05). The N-SMI group had more bacteria labeled with green bars belonging to the phylum Firmicutes. In contrast, the L-SMI group had more bacteria belonging to the phyla Bacteroidetes and Proteobacteria. (**b**). The bar graph of LDA score calculated by LEfSe. The bar graph shows the LDA score for each bacterium detected by the same method as in Fig. 2a. LEfSe is ranked according to the effect size and associated with the class with the highest median value. This analysis was calculated in LEfSe using the default settings (alpha value of 0.05) and the p-values for all bacteria were less than 0.05.

The bacteria illustrated in Fig. 2 show a significant difference in relative abundance between the two groups. Bacteria shown with green bars are higher in relative abundance in the N_SMI group than in the L_SMI group. The relative abundance of Proteobacteria and Bacteroides was higher in the L_SMI group than in the N_SMI group. Conversely, the relative abundance of bacteria belonging to the phylum Firmicutes was higher in the N_SMI group than in the L_SMI group. At genus level, the relative abundance of *Coprobacillus*, *Catenibacterium* and *Clostridium* were lower and *Bacteroideswer* higher in in the L_SMI group than in the N_SMI group (p < 0.05).

### Comparison of predictive functional profiling via Phylogenetic Investigation of Communities by Reconstruction of Unobserved States 2 (PICRUSt2; predictive function genes)

**Figure 3:**
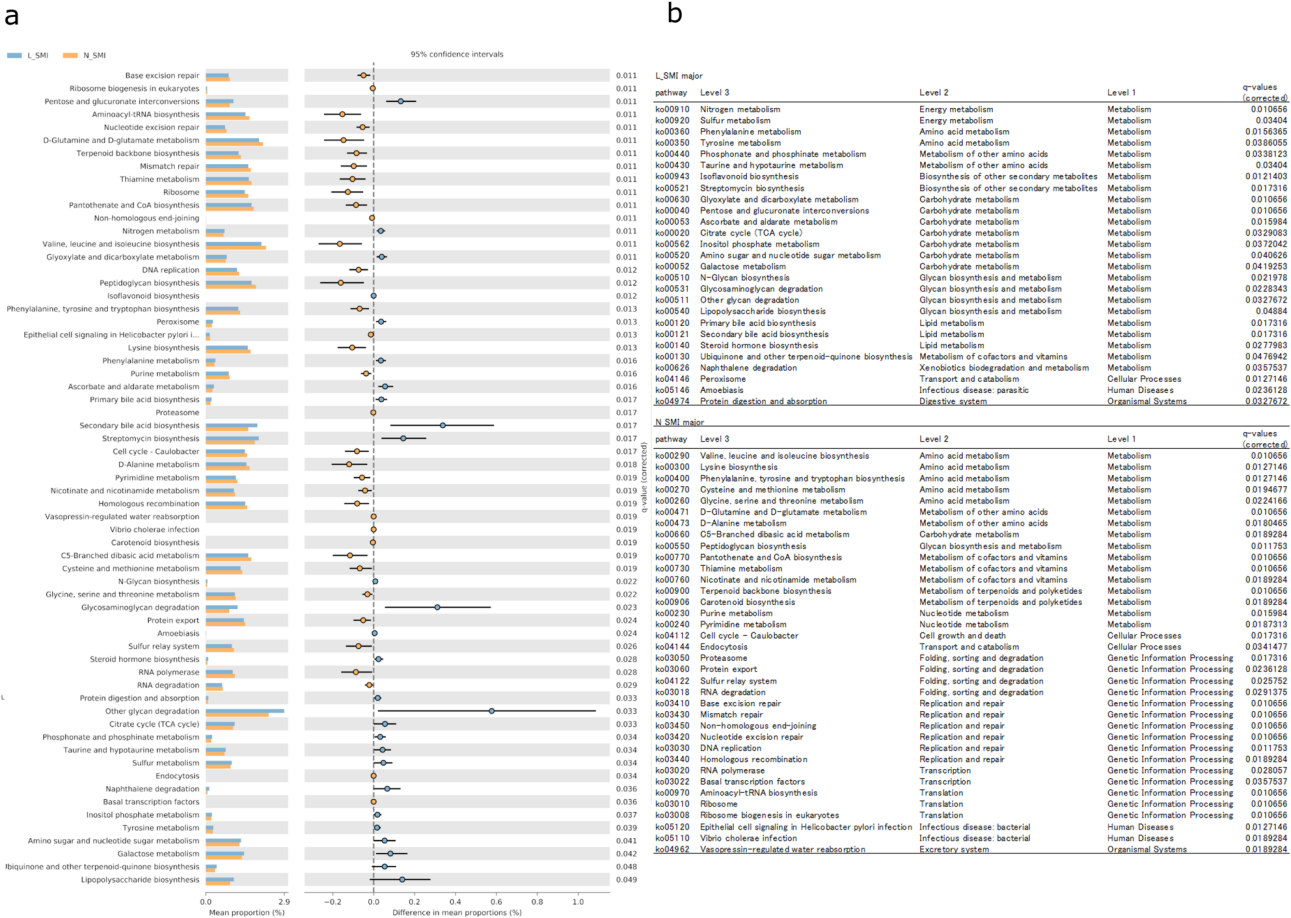
(**a**). Differences in the predictive functional profiling between the two groups. The program PICRUSt2 can predict the role of bacteria identified from 16srRNA, including their function, by referring to a database (KEGG). Each of these predicted genes was classified according to the KEGG category. Among them, the categories with significant differences between the two groups are shown (q-value < 0.05). (**b**). The category detected with significant deference were sorted by level 2 of the KEGG category. The L-SMI group has many more pathways related to carbohydrate metabolism and less related to amino acid metabolism. In terms of amino acid metabolism, genes associated with valine, leucine, and isoleucine synthesis were the most abundant in the N-SMI group. Differences in function and metabolism suggest that the metabolites produced by the microbiome may differ.

PICRUSt2 is a method to predict functional genes from 16 s rRNA data by referring to KEGG. This method provides information on the enzymes and bacterial skeletons as well as bacterial names. The results were calculated based on each K number amount. Figure 3a shows only those categories with a significant difference between the two groups (q-value < 0.05). The results were categorized according to metabolism and roll at each level in Fig. 3b. A total of 63 categories have been identified with significant differences. Each pathway belonged to a category by three levels, according to KEGG analyses. Among the pathways detected, the number of categories belonging to metabolism was the highest. The L-SMI group was characterized as follows: 1) the abundance of pathways classified as nitrogen metabolism was higher among metabolism categories. However, genes associated with amino acid metabolism were low, while some of those associated with carbohydrate metabolism were high, which suggests that more bacteria obtain energy from carbohydrates than amino acids. In addition, although enough nitrogen metabolism was evident, a few genes were associated with amino acids, which may indicate that nitrogen is converted to other than amino acids. 2) The gene levels associated with valine, leucine, and isoleucine biosynthesis were lower with significantly different than the N-SMI group. 3) High levels of ‘LPS biosynthesis’ involved LPS, its precursors, and metabolites, which are often synthesized in the cell walls of gram-negative bacteria. These nearest sequenced taxon index distributions were calculated automatically in PICRUSt2 and the average of all samples was 0.0397 ± 0.01998.

### Comparison of genes for enzymes related to amino acid synthesis

**Figure 4:**
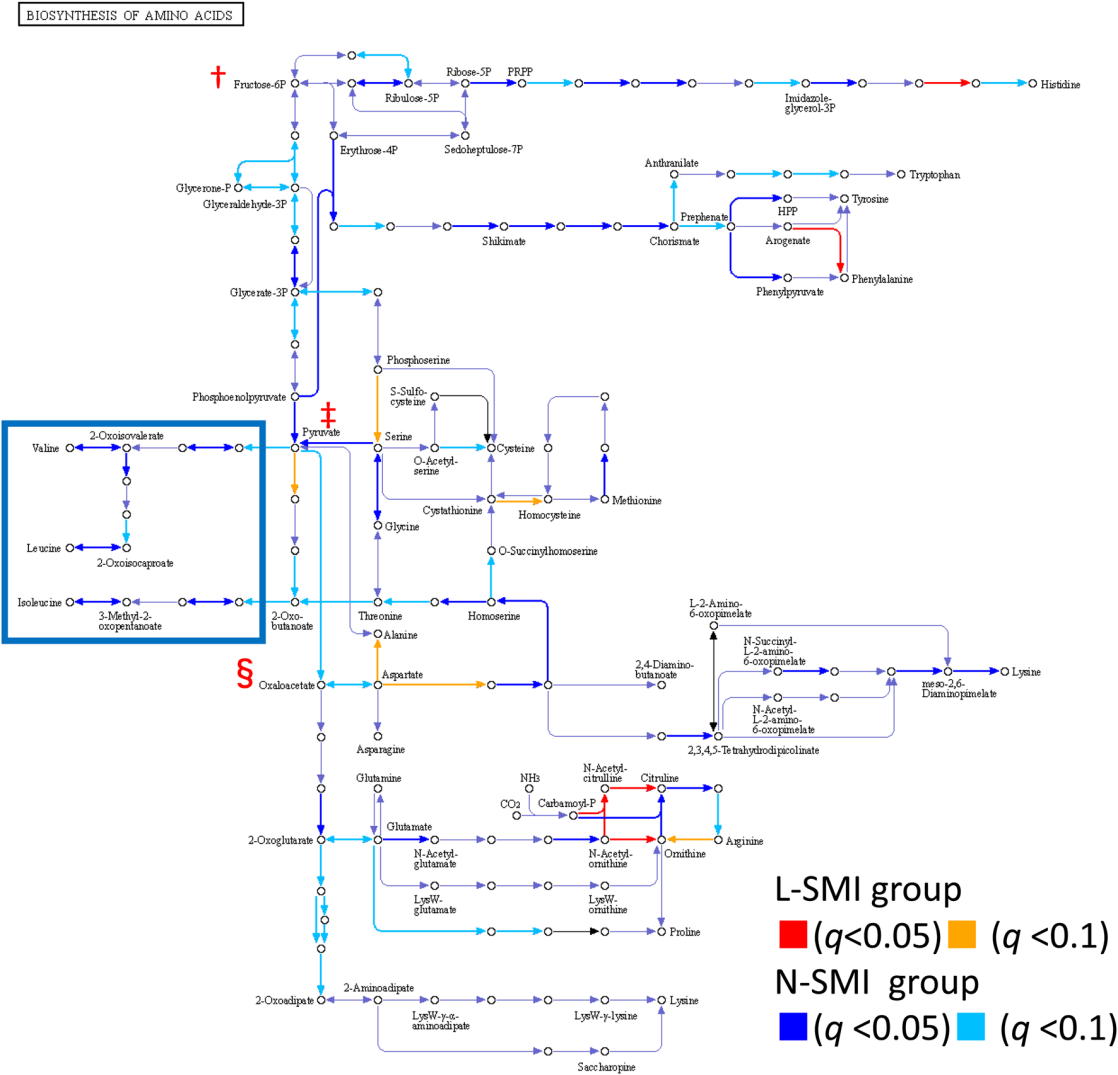
Mapping of functional genes for amino acid synthesis. The genes predicted using PICRUSt2 are numbered with K numbers. Each K number related to amino acid synthesis refers to an enzyme. The ‘○’ in the pathway diagram shows the metabolite, and the ‘ → ’ indicates the metabolic enzyme. The amount of the gene for the enzyme in each patient's microbiome was compared between the two groups. The pathway with a q-value less than 0.1 is colored. Multiple blue or light blue arrows show that the N-SMI group had more enzymes associated with each amino acid synthesis compared to the L-SMI group. The pathways related to valine, leucine, and isoleucine were more abundant in the N-SMI group than the L-SMI group. Metabolic pathways are surrounded by blue squares. For better understanding, the important factors in amino acid synthesis, fructose-6-phosphate (†), pyruvate (‡), and oxaloacetic acid (§), are indicdated with a marked focus.

The most categories belonged to metabolism, as shown in Fig. 3b. Certain categories belonging to metabolism included enzymes associated with amino acids. KEGG mapping for amino acid synthesis is shown in Fig. 4. The corresponding enzymes were identified from the K numbers detected using PICRUSt2. Fructose-6-phosphate (†), which is critical in amino acid synthesis, is shown in the upper left corner of Fig. 4. Pyruvate (‡) and oxaloacetic acid (§) are shown downstream of this pathway. Each amino acid is synthesized by multiple enzymes. The various pathways shown in blue indicate that the L-SMI group may have fewer amino acid enzymes than the N-SMI group. In particular, the N-SMI group had significantly more predicted genes involved in the valine/leucine/isoleucine pathway than the L-SMI group, as indicated by the blue squares. The detailed pathway of valine, leucine, and isoleucine biosynthesis was also shown in Supplemental Figure.

## Discussion

Sarcopenia is a progressive and generalized skeletal muscle disorder caused by various factors such as systemic inflammation, insulin resistance, and myokine and adiponectin dysregulation ^10^. Patients with chronic liver disease are more affected by these factors since their energy metabolism and amino acid synthesis are weaker than those of healthy individuals ^11^. In patients with cirrhosis, sarcopenia can increase sepsis mortality and risk of hepatic encephalopathy, and prolonged hospitalization following liver transplantation ^12^. Furthermore, Montano-Loza et al. reported that combining the MELD score with sarcopenia can more accurately predict mortality ^13^. Therefore, sarcopenia is an independent prognostic factor of MELD.

Recently, the link between muscle and microbiome　has attracted attention. Abnormal microbiome can increase intestinal mucosal permeability, thereby causing bacterial components, including LPS, to flow more easily into the bloodstream, which activate the inflammatory response and increase circulating pro-inflammatory cytokines such as interleukin-6 and tumor necrosis factor-α ^14^. LPS has been suggested to induce systemic inflammation and may be a progression-related factor in sarcopenia, and that increased short-chain fatty acid (SCFA) production by the microbiome may have anti-inflammatory effects and a positive impact on human skeletal muscle mass and body function ^15^. SCFAs, especially butyrate, reportedly exert a significant effect on skeletal muscle cell function by promoting mitochondrial activity ^16^. Increases in portal blood LPS and decreases in SCFA-producing bacteria are more likely to occur in cirrhosis ^17^. SCFA, LPS, and systemic inflammation caused by microbiome are recognized as the gut-muscle axis and influence muscles ^2^. BCAAs are associated with muscle mass. The Muscle-Gut-Brain Axis ^18^ and the Kidney-Gut-Muscle Axis ^19^ are being recognized according to the characteristics of each disease.

Differences in the microbiome between patients with or without sarcopenia are important but it have not yet been reported enough. Lin Kang et al^5^. compared 11 patients with sarcopenia and 16 Possible sarcopenia with 60 Healthy control. They showed that sarcopenia patients had lower Chao1 and observed species diversity. In particular, they also showed a lower relative abundance of Firmicutes. Our study also showed that Firmicutes were lower in the L-SMI group compared to the N-SMI group. In addition, in difference to Lin Kang et al., we compared patients with liver disease and the results showed that the ratio of Bacteroidetes was also significantly higher. Our study suggests that the microbiome influences sarcopenia through LPS and BCAA synthesis. The Firmicutes to Bacteroidetes ratio was lower in the L-SMI group than N-SMI group. LPS is found in the cell walls of gram-negative bacteria. The abundance of LPS biosynthesis protein was higher in the L-SMI group compared to the N-SMI group due to the abundance of Bacteroidetes, gram-negative bacteria, and fewer Firmicutes, a gram-positive bacterium, in the L-SMI group. The mucosal barrier prevents LPS from entering the bloodstream even in the presence of high levels of LPS in the normal intestinal tract. However, in systemic diseases such as chronic liver disease, LPS is more likely to flow into the bloodstream due to decreased SCFA and disruption of the intestinal barrier mechanism^20^.

We also showed that the bacteria in the L_SMI group contained a smaller proportion of enzymes associated with BCAA synthesis. It has recently been shown that microbiome synthesized amino acids might be associated with muscle mass ^21^. Bacteria are diverse in the microbiome, and many include enzymes associated with nitrogen metabolism. The L-SMI group showed higher nitrogen metabolism potential in its microbiome relative to the N-SMI group but lower levels of valine, leucine, and isoleucine biosynthesis. Considering the level 2 category, the L-SMI group contained few amino acid metabolism-related genes and high number of genes associated with carbohydrate metabolism. This result may indicate that a high proportion of bacteria in L-SMI group use carbohydrates for energy metabolism. Regarding amino acid metabolism, the most significant differences were found in valine, leucine, and isoleucine biosynthesis. The category of nitrogen metabolism includes the central role of the pathway in the synthesis of ammonia from nitrate.

Xiao et al^22^. showed that both nitrogen metabolism and valine, leucine, and isoleucine biosynthesis upregulation were microbiome characteristics in patients with cirrhosis. In contrast, our results showed that valine, leucine, and isoleucine biosynthesis was lower in the L-SMI group compared to the N-SMI group. This may be an important feature of sarcopenia in patients with chronic liver disease since ‘valine, leucine, and isoleucine biosynthesis does not decline in healthy individuals until old age ^23^. Ponziani FR et al. ^6^ also showed that the microbiome of chronic liver disease patients with sarcopenia has low gene levels of enzymes associated with BCAA synthesis. These results and ours are in line in several categories, including D-glutamine and D-glutamate metabolism, pentose and glucuronate interconversions, and D-glutamine and D-glutamate metabolism. However, we also performed KEGG mapping since the KEGG analysis alone cannot assess whether the activity on BCAA synthesis is high since the category includes both BCAA synthesis and degradation enzymes. Figure 4 demonstrates the predicted abundance of these enzymes suggesting that the N-SMI group is more likely to synthesize valine, leucine, and isoleucine. This suggests that due to the low levels of microbiome-derived BCAAs, patients with sarcopenia may have lower amino acid uptake than non-sarcopenic patients, even if they consume a similar diet.

Although it is controversial whether BCAAs contribute significantly to the systemic BCAA pool, several reports have shown an association between blood BCAA concentrations and the microbiome^4^. Pedersen et al.^24^ reported that the number of genes associated with BCAA synthesis encoded in bacteria correlated with the concentration of BCAAs in the blood and affected insulin resistance. Dhakan et al. ^25^, through multi-omics approaches, reported that BCAA-encoding genes positively correlated with BCAA in the blood. The characteristics of their bacteria showed a higher Bacteroidetes to Firmicutes ratio (*p* = 0.002), and several bacteria were associated with it. Serum BCAA is correlated with SMI in patients with liver cirrhosis ^26^. These studies and our results suggest that high levels of genes associated with BCAA biosynthesis may affect the BCAA pool and muscle mass.

BCAAs are important for both sarcopenia and chronic liver disease. In investigating sarcopenia, previous studies have reported that protein intake did not improve muscle mass^27,28^, while amino acid intake, including leucine, improved muscle atrophy^29^. High-quality amino acid intake is more important than protein intake, especially in patients with chronic liver disease, as they are unlikely to synthesize amino acids from proteins due to anabolic resistance^30^. As a treatment for anabolic resistance, proteins are insufficient to replace the amount of nitrogen consumed; therefore, late evening snacks such as BCAAs are required ^31^.

We hypothesize that bacteria play an important role in amino acid synthesis in the human body. Plants take up nitrogen from the soil via the microbiome. The intestinal tract contains endogenous nitrogen, waiting to be excreted, where microbiome recycles some it to synthesize amino acids or ammonia. We have shown several differences in the KEGG category, of which we have only mentioned LPS and BCAA synthesis. However, the microbiome has many other roles, and further analysis is warranted.

We have shown that chronic liver disease patients with sarcopenia may have differences in microbiome, increased LPS and decreased BCAA synthesis capacity, even in the percentage of patients with severe cirrhosis were low. However, this study has several limitations. First, we were unable to measure NH_3_ and LPS, although we have cited reports on the association between microbiome and blood LPS and BCAA. Second, we could not perform multivariate analysis on the effect of covariates (Etiology was significantly different and HCC was borderline different between the two groups) due to the small number of patients enrolled. Sarcopenia is usually diagnosed by measuring grip strength and overall muscle mass. Prospective observational studies, including grip test, metabolomics and mass spectrometry, may be necessary to address these limitations.

In conclusion, patients with low muscle mass in chronic liver disease had a significantly lower Firmicutes to Bacteroidetes ratio and may contain more gram-negative bacteria and more LPS. Their microbiome contains fewer enzymes that synthesize intestinal nitrogen into BCAAs. These results provide insights into the development of prebiotics/probiotics that can provide a gut environment for muscle mass growth via the microbiome.

## Materials and methods

### Study design

In this single-center study, 69 patients with HCV antibody/HBs antigen-positive chronic liver disease were classified into two groups based on their SMI and compared for their microbiome and its predictive functional profiling. SMI was calculated based on the muscle mass area at the L3 vertebral level using the SYNAPSE VINCENT software (Fujifilm Medical, Tokyo, Japan). The microbiome was sequenced via MiSeq, and the results were compared with LEfSe^32^ and PICRUSt2^33^.

The Research Ethics Committee of Nagoya University Hospital (protocol number 2015–0420, August 30, 2016) approved our research, and written informed consent was obtained from all patients before enrolment in accordance with the Declaration of Helsinki. This study was registered in the University Hospital Medical Information Network Clinical Trials Register (UMIN ID: 002,020,269). All clinical and stool sample information was anonymized, and a database was constructed.

### Patient selection

Japanese patients with HCV antibody/HBs antigen-positive chronic liver disease and cirrhosis aged 50–79 years were enrolled in the NAGOYA gut microbiota database between June 2016 and March 2020 for inclusion in the study. The patients enrolled in this study did not have a history of antibiotic or immunosuppressive use within one month, gastric or colorectal surgery, or active infection. Patients were classified into the normal SMI group (N-SMI, n = 44) and low SMI group (L-SMI, n = 25) according to their SMI values, and were followed up by a hepatologist. At the time of stool sample collection, blood tests and imaging studies, including ultrasound (US), computed tomography (CT), or magnetic resonance imaging (MRI), were also conducted. Sample collection was followed up with liver function tests every 3–6 months. HCC was defined as the presence of hypervascularity on CT, MRI, or contrast-enhanced US. At least two types of imaging were performed and HCC was diagnosed by at least two hepatologists. Patients answered questionnaires regarding defecation frequency and exercise habits.

### Measurement of SMI

Muscle mass was also estimated by calculating the muscle mass area at the level of the L3 vertebrae using SYNAPSE VINCENT^34^. Individual sections of the CT scan at the L3 vertebral level were isolated, and areas of the lumbar muscles, paraspinal column, and abdominal wall were outlined. Using a range of Hounsfield units where the muscle tissue absorbs x-rays (− 29, + 150), the cross-sectional area of the above mentioned muscles was semi-automatically quantified, leading to an estimate of the total cross-sectional area of the abdominal skeletal muscle at the L3 level. The estimated muscle mass area was normalized to the height to calculate the SMI. The cut-off values for SMI were 42 cm^2^/m^2^ for men and 38 cm^2^/m^2^ for women according to the Japan Society of Hepatology guidelines for sarcopenia in liver disease (1st edition)^35^. According to the former guideline, it was reported as ” These cut-offs are 42  cm^2^/m^2^ for men (AUC, 0.83; sensitivity, 89%; specificity, 57%) and 38 cm^2^/m^2^ for women (AUC, 0.85; sensitivity, 95%; specificity, 96%)”.

### Sample collection and 16S rRNA gene sequencing

Sample collection and sequencing were performed as previously described^36^. Samples were collected at home (n = 18) or the hospital (n = 51); no significant difference in the collecting method between N-SMI and L-SMI groups was observed (*p* = 0.399, Pearson's chi-square test). The difference between the two sampling methods has been reported to have no impact on the microbiome results^37,38^. Isolated DNA using the DNeasy PowerSoil Kit (Qiagen, Hilden, Germany) was amplified using universal primers (forward: 5’-TCGTCGGCAGCGTCAGATGTGTATAAGAGACAGCCTACGGGNGGCWGCAG-3’ and reverse: 5’-GTCTCGTGGGCTCGGAGATGTGTATAAGAGACAGGACTACHVGGGTATCTAATCC-3’) to target the V3–4 regions of bacterial 16S rRNA. Sequencing data were obtained using the MiSeq Reagent Kit v3 (Illumina, San Diego, CA, USA), with 2 × 300 reads and 600 cycles for microbial analysis.

The raw paired-end reads from Miseq were analyzed using QIIME2 (version 2019.11 https://docs.qiime2.org/2020.11/) pipeline^39^. DADA2 was used to assess the quality of the reads which included filtering, trimming, denoising, dereplicating, merging of the forward, and reverse strands as well as removing chimeras^40^. We obtained a total of 7,284,384 demultiplexed sequence counts (paired-end reads) with 3162 features identified. Amplicon sequence variants (ASV) were aligned using mafft-plugin which was subsequently used for the fassttree2-plugin which was needed for the diversity analysis^41,42^. The median sequencing depth was 48,953 and Minimum frequency was 6,356. Taxonomy was assigned to the 16S data using Greengenes 13_8(https://greengenes.secondgenome.com/) . Differences in the abundance of bacteria was calculated using LEfSe^32^ and the predicted metagenome functions were calculated using the PICRUSt2^32,33^.

### Statistical analysis

The two groups were compared using chi-square or Fisher's exact test for categorical variables and Mann–Whitney *U* test for continuous variables as appropriate. The data were analyzed using IBM SPSS Statistics version 24.

Microbiome comparisons and diversity were visualized and statistically analyzed using MicrobiomeAnalyst^43^. Alpha diversity was calculated using Shannon index, Chao1, and observed species. The analysis was performed with the default settings, and the taxonomic level was set as the feature level. Alpha diversity and bacteria at the phylum level were compared using Mann–Whitney U. Beta diversity was calculated using ANOSIM (bray–curtis index), a non-parametric test as a statistical method, and the taxonomic level was selected as Feature and Genus level. To compare bacteria to genus level in the two groups, LEfSe was used with default settings (α = 0.05). The p-value of the detected bacteria was confirmed to be less than 0.05.

The PICRUSt2 results were calculated using STAMP ^44^, and the two groups were compared using White’s non-parametric *t*-test. Storey’s FDR was selected as multiple test correction methods to adjust significance for multiple hypothesis testing. The predicted abundance of the gene by K number was also compared using the same methods. The adjusted p-values are shown as q-values because Storey's FDR was used.

### Ethics approval

The Research Ethics Committee of Nagoya University Hospital (protocol number 2015–0420, August 30, 2016) approved our research.

### Consent to participate

Written informed consent was obtained from all patients before enrolment in accordance with the Declaration of Helsinki.

### Consent for publication

We received the permission to publish when we obtained informed consent.

## Supplementary Information

Supplementary Information 1.

Supplementary Information 2.

## Data Availability

The data that support the findings of this study are available from Department of Gastroenterology and Hepatology, Nagoya University Graduate School of Medicine but restrictions apply to the availability of these data, which were used under license for the current study, and so are not publicly available. Data are however available from the authors upon reasonable request and with permission of Department of Gastroenterology and Hepatology, Nagoya University Graduate School of Medicine.

## Abbreviations

HCC: Hepatocellular carcinoma
LPS: Lipopolysaccharide
SMI: Skeletal muscle mass index
BCAA: Branched-chain amino acid
LEfSe: Linear discriminant analysis effect size
PICRUSt2: Phylogenetic Investigation of Communities by Reconstruction of Unobserved States 2
KEGG: Kyoto Encyclopedia of Genes and Genomes
SCFA: Short-chain fatty acid
