# Supplementary material for: Patients with low muscle mass have characteristic microbiome with low potential for amino acid synthesis in chronic liver disease
Source: Sci Rep. 2022 Mar 7;12:3674. doi: 10.1038/s41598-022-07810-3 (PMC8901651; doi:10.1038/s41598-022-07810-3)
Supplement: Supplementary file 1 — Supplementary Information 1. [file 41598_2022_7810_MOESM1_ESM.pdf]

Supplemental figure

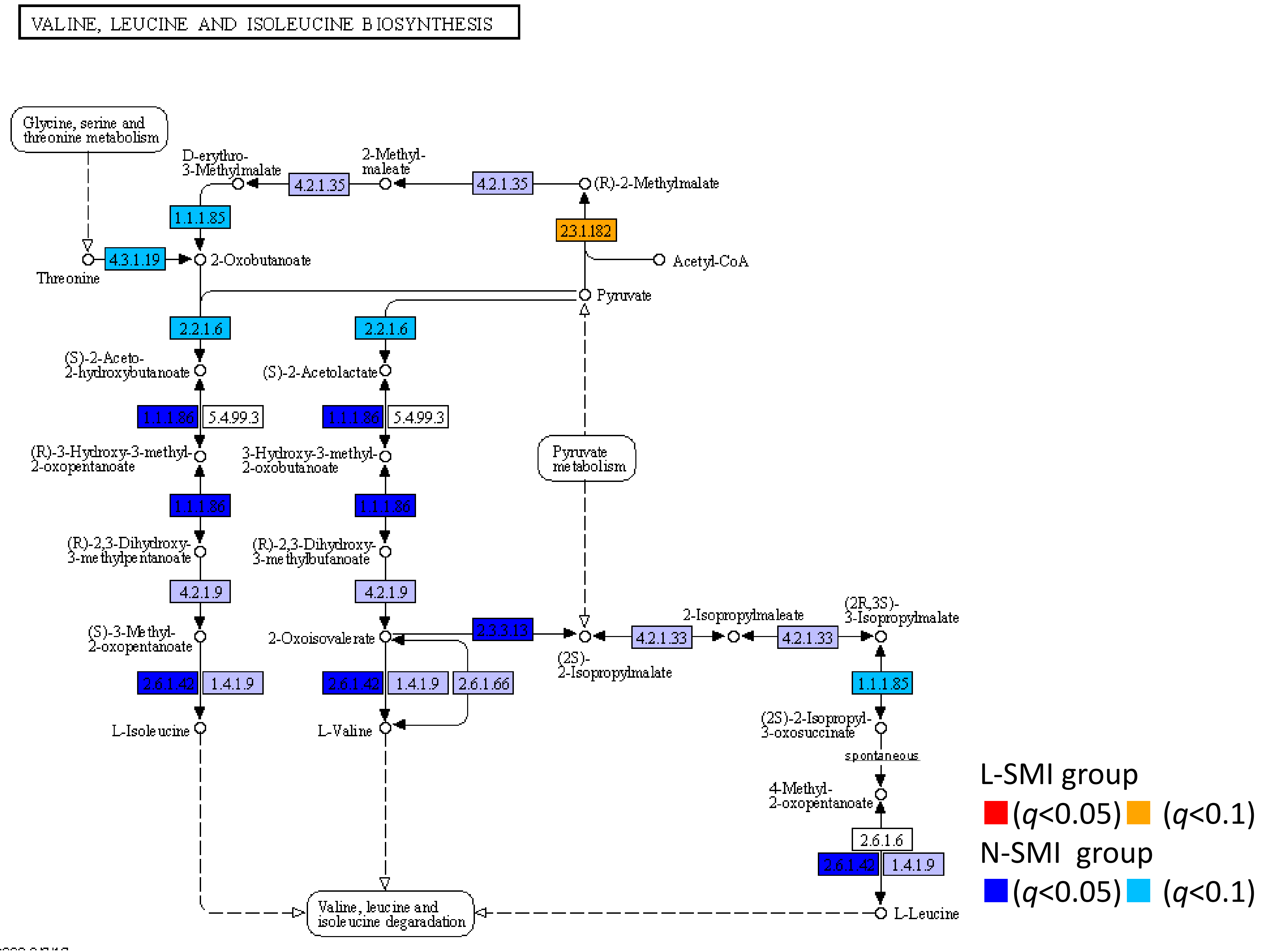

Supplementary Figure. Mapping of functional genes for amino acid synthesis.

Comparison of predicted genes in the KEGG pathway of " Valine, leucine and isoleucine biosynthesis". We have shown in Figure 4 that enzymes related to amino acid biosynthesis are different between L-SMI and N-SMI groups. Among them, we think BCAA synthesis is the most important. Therefore, we further evaluated the KEGG pathway of "Valine, leucine and isoleucine biosynthesis". According to this pathway, the N-SMI group had more predicted genes for many enzymes related to BCAA synthesis than the L-SMI group.

title: Chronic liver disease patients with low muscle mass have characteristic microbiome and predictive functional profiling of amino acid synthesis

Author: Kenta Yamamoto, Yoji Ishizu, Takashi Honda, Takanori Ito, Norihiro Imai, Masanao Nakamura, Hiroki Kawashima, Yasuyuki Kitaura, Masatoshi Ishigami, Mitsuhiro Fujishiro

journal: Scientific reports
